# What should a quantitative model of masking look like and why would we want it?

**DOI:** 10.2478/v10053-008-0011-6

**Published:** 2008-07-15

**Authors:** Gregory Francis

**Affiliations:** ^1^Psychological Sciences, Purdue University, West Lafayette^2^Brain Mind Institute, École Polytechnique Fédérale de Lausanne (EPFL), Lausanne

**Keywords:** backward masking, dynamic vision, modeling

## Abstract

Quantitative models of backward masking appeared almost as soon as computing technology was available to simulate them; and continued interest in masking has lead to the development of new models. Despite this long history, the impact of the models on the field has been limited because they have fundamental shortcomings. This paper discusses these shortcomings and outlines what future quantitative models should look like. It also discusses several issues about modeling and how a model could be used by researchers to better explore masking and other aspects of cognition.

##  

Backward masking refers to reduced visibility of a target stimulus when it is followed by a mask stimulus. The conditions under which masking occurs, and some special properties and uses of backward masking, are well summarized in other papers in this issue (Breitmeyer this volume, Enns, & Oriet, this volume). This paper looks at the status of quantitative models, considers some issues and limitations about such models, and then explores how to proceed in a way that will improve the study and use of backward masking.

Studies of masking often vary the timing between the target and mask stimulus. A measure of target visi-bility plotted against the stimulus onset asynchrony (SOA) between the target and mask is called a masking function. Empirical work typically finds two types of masking functions, referred to as Type A and Type B. A Type A masking function is shown in Figure 1a. The visibility of the target is minimized for common onset of the target and mask (SOA = 0). As the SOA increases, the target becomes more visible. A Type B masking function is shown in Figure 1b. The target is easily visible for common onset of the target and mask stimuli, but becomes less visible as the SOA increases. After reaching a minimum of visibility (maximum of masking) at some intermediate SOA, target visibility increases. Whether Type A or Type B masking is produced depends on the target, mask, experimental task, and conditions of the experiment, as is discussed in other papers in this issue (Breitmeyer this volume, Bridgeman this volume, Herzog, this volume).

**Figure 1.**
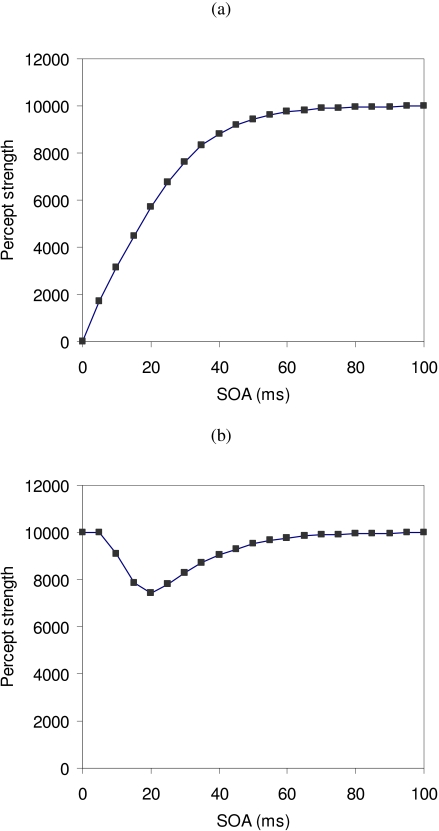
A plot of target percept strength against SOA between the target and mask stimuli is called a masking function. (a) A Type A masking function occurs when the target percept strength increases with SOA. (b) A Type B masking function occurs when the target percept strength decreases then increases with SOA.

Scholarly papers on backward masking often describe it as mysterious, paradoxical, or surprising. These claims about backward masking are of two types. First, it is surprising to some researchers that a trailing mask can affect the visibility of the leading target. Indeed, the phenomenological appearance of the target-mask sequence is sometimes that only the mask is presented. This result is surprising for some views of neural processing that supposes information proceeds in a feed forward manner. In some such views, the earlier target information would always be at a neural location where the mask information was not. In such a view, masking requires the mask information to lead ahead in space (or backward in time) to interfere with the target percept.

The second mysterious or paradoxical property of masking is that Type B masking should ever exist. It might seem that whatever interference might be caused by the mask, it should be strongest when the target and mask maximally overlap in time. Type B masking indicates that this is not always true. Instead, the strongest masking sometimes occurs when the mask follows the target by tens of milliseconds.

These properties of backward masking may, indeed, have been mysterious, paradoxical, or surprising 70-100 years ago when they were discovered (Alpern, 1953; Stigler, 1910; Werner, 1935), but the mystery is no longer a motivation to study masking. Studies of quantitative models reveal that these properties of masking are quite easy to explain in a variety of ways. There are, in fact, over a dozen models that have been applied to backward masking conditions, and most can explain the appearance of both Type A and Type B masking functions. The oldest models are over 35 years old, which suggests that the mystery, surprise, and paradox of backward masking persist only for those who do not know of the modelling work.

One of the earliest computational models in psychology was proposed by Weisstein (1966, 1972) to study aspects of backward masking. At about the same time Bridgeman (1971, 1978, this volume) showed that masking was a natural property of a system of recurrent lateral inhibition. Anbar and Anbar (1982) demonstrated that a model of brightness perception showed Type B masking when extended to the temporal domain. Reeves (1982) introduced a probabilistic model that explains some relationships between masking functions and perceptual experiences of integration and success. During much of the 1980s, interest in masking waned generally, and there were fewer new models. Interest was renewed in the 1990s and models soon followed. Öğmen (1993) and Purushothaman, Öğmen, and Bedel (2000) proposed a neural network model that was conceptually linked to Weisstein’s model. Bachmann (1994) included equations to emulate aspects of his perceptual retouch model. Francis (1997) investigated the dynamics of Grossberg and Mingolla’s (1985) model of visual perception and found that it matched a variety of masking data.

Since the turn of the century, there have been even more models. Francis (2000, 2003a, 2003b) identified a variety of computational systems that could account for many properties of masking. Di Lollo, Enns, and Rensink (2000) proposed the Computational Model of Object Substitution (CMOS), which nicely fit their experimental findings on common onset masking. Herzog, Ernst, Etzold, and Eurich (2003) found that many properties of masking could be accounted for with a simple network of Wilson-Cowan equations (see also Hermens & Ernst, this volume). Bugmann and Taylor (2005) found that Type B masking was produced by a hierarchical pyramid structure of visual processing. Francis and Cho (2005, 2007) identified a simple model that uses one of the computational systems identified in Francis (2000) . Bowman, Schlaghecken, and Eimer (2006) used a model of masking to explain some aspects of subliminal priming.

Clearly, there are many different models that account for properties of backward masking. Significantly, many of these models were originally designed for entirely different reasons. This includes the models of Bridgeman (1971) , Anbar and Anbar (1982) , Öğmen (1993) , Francis (1997) , Herzog et al. (2003) , and Bugmann and Taylor (2005). Such models demonstrate that many properties of backward masking are a natural part of visual processing.

Why are there so many different models of backward masking? Considering this question reveals some important issues about modelling and backward masking. The first answer is that there are so many models of masking because there is no general theory of visual perception that might place constraints on the structure and properties of models. Without a general theory, it is fairly easy to introduce a new model and argue against other models.

Second, some aspects of masking, such as the existence of Type B masking (Breitmeyer & Öğmen, 2000) or common onset masking (Di Lollo et al., 2000) have been described as difficult to explain. Modellers are drawn to challenges and so explore whether their model can account for the empirical results. Success is often reported, but it is often less because of the details of the model and more because many of the models explain aspects of masking with similar basic principles. For example, Francis and Cho (2005) show how a small system with four equations can produce a Type B masking function. Bugmann and Taylor (2005) used a system with 341 equations to also produce a Type B masking function. There are many important differences between the models and there are differences in the quantitative values of their masking functions. Nevertheless, both models produce a Type B masking function for essentially the same reasons. There are many different models of masking, in part, because researchers end up repeating the same basic principles in a variety of models.

Such repetition is worthwhile. The model proposed by Francis and Cho (2005) demonstrates one of the simplest systems that can produce a Type B masking function. In contrast, the model of Bugmann and Taylor (2005) demonstrates that the same basic principle robustly applies even when it is embedded in a much more complicated system. There is value to both kinds of implementations of the principle.

On the other hand, this kind of repetition is not often recognized as repetition. The models of Weisstein (1972) and Bridgeman (1971) have often been considered as very different models, but Francis (2000) showed that both models operate with a common basic principle. Likewise, Di Lollo et al. (2000) introduced their model in part because they claimed other models could not account for their data. However, Francis and Hermens (2002) demonstrated that many models could account much of their experimental data. In general, models that look very different may still operate with the same basic principles.

## TESTING MODELS OF BACKWARD MASKING

 Many experimentalists seem to believe that the best model is the one with the fewest parameters; a variation of Occam’s razor. However, this view is too narrow. Consider, for example, a comparison of the Francis and Cho (2005) and Bugmann and Taylor (2005) models. Both explain the general shape of Type B masking. Which model is better? A comparison of parameters would seem to favour the model of Francis and Cho, which has very few parameters, over the model of Bugmann and Taylor, which has thousands of parameters. If one just wants to talk about ways of producing the Type B masking function, then this may be a reasonable conclusion. But we are less interested in masking functions than in visual perception in general. In this regard both models are so far from the truth (the human visual system would need billions or possibly trillions of parameters to be characterized) that the question of which model is better is not likely to be settled by counting the number of parameters. 

The current state of modelling backward masking has both pros and cons. The pros include a rich set of models that operate at many different levels. Such variety indicates that there is an interest in developing models of masking. The cons include that all of the models are so simple that they cannot possibly be correct. In this regard, it is very difficult to test models. Indeed, it is not at all difficult to find shortcomings in any of the quantitative models. For example, none of the models deal with depth perception, colour vision, short term memory, or human decision making. Making progress in modelling depends not so much on identifying flaws in the models, but in identifying those particular flaws that either force a complete rejection of a model or suggest how to modify the model.

**Figure 2.**
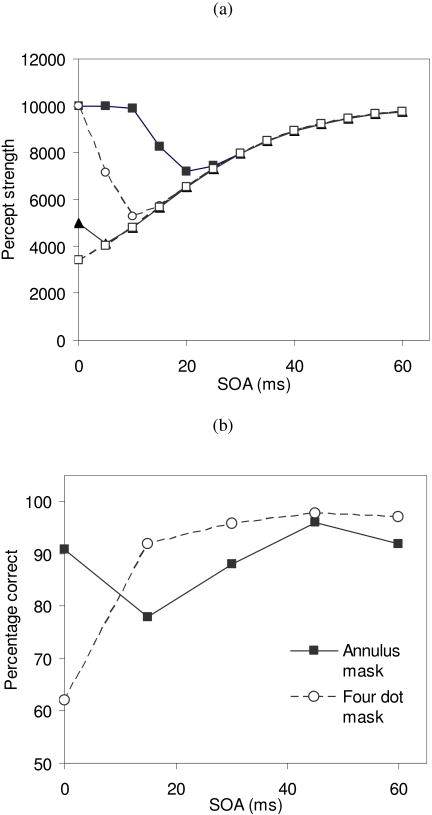
(a) Simulation results from the model of Francis and Cho (in press) show that the shape of the masking function is related to masking strength. Type A functions occur for strong masks and Type B functions for weaker masks, and the curves never cross. (b) An experimental study in Francis and Cho (in press) varied the spatial shape of the mask. The shape of the masking function is not related to masking strength and the curves cross.

Francis and Herzog (2004) recently identified one such flaw. There is a notable characteristic of almost all of the models regarding how they produce Type A and Type B masking functions. All of the models predict that the shape of the masking function is connected to the overall strength of masking. Namely, strong masks should produce Type A masking functions, while weak masks should produce Type B masking functions. Figure 2a shows masking functions generated by the model of Francis and Cho (2007) for masks of different intensities. The Type B masking functions always lay above the Type A masking functions at each SOA, and this effect is a property of many different models of backward masking (Francis & Herzog, 2004). Thus, all of these models predict that if the target and task are held fixed, then variations in the mask (intensity, duration, or shape) could vary the shape of the masking function from Type A to Type B, but only such that the masking function curves do not intersect.

We have now identified several circumstances where this prediction does not hold (Francis & Cho, 2007; Francis & Herzog, 2004). Figure 2b combines data from two experiments in Francis and Cho (2007) , where the target and task were always the same (identify the orientation of a half disk target among three full disk distracters), but the spatial shape of the mask varied. The main finding is that variations in the spatial shape of the mask lead to Type A or Type B masking functions, but that these masking function shapes were not related to the overall strength of masking.

This data presents a significant problem for all of the current models. There is no variation of parameters that will allow the models to match this experimental finding. There needs to be entirely new kinds of models with properties quite different from the current models.

One of the key problems with the current models is that they do not have a sufficiently rich representation of the spatial properties of the target and mask stimuli (Herzog, this volume). For many of the models, the representation of the mask is simply a numerical value that changes over time. This is explicitly the case for the models by Weisstein (1972) , Anbar and Anbar (1982) , Bachmann (1994) , Di Lollo et al. (2000) , Francis (2003a) , and Francis and Cho (2005) . Even for models that include a spatial representation of stimuli, the calculations of masking often reduce the mask’s effect on the target to a single numerical value. Francis (2000) showed that this was the case for the recurrent lateral inhibition model of Bridgeman (1971, 1978), and a similar conclusion appears to be true for the models of Francis (1997) , Purushothaman et al. (2000) , Herzog et al. (2003) and Bugmann and Taylor (2005).

The significance of this property is that a variation in the spatial shape of the mask, as in Figure 2b can only lead to a differing magnitude (or duration) of the corresponding mask’s effect in the model. Thus, advancement of the models requires a substantial elaboration of the spatial aspects of the models. Interestingly, Weisstein (1972) long ago recognized the need for models to include spatial as well as temporal properties of masking. Indeed, it is obvious that any attempt to build a model of visual perception that does not include spatial vision is missing an important part of the story.

There are two primary reasons why it has taken over 30 years to return to Weisstein’s observation that models of backward masking must combine both spatial and temporal aspects of visual perception. First, the current models, even with their limited spatial representation of stimuli, have successfully accounted for many properties of backward masking. Second, computing resources have not generally been available to build models of visual perception that incorporate both space and time. Even the computer simulations with current models sometimes take days or weeks (Francis, 1997; Purushothaman et al., 2000) to carry out key simulations. Models that include a richer spatial representation (e.g., Cao & Grossberg, 2005; Grossberg, 1997; Itti, Koch, & Niebur, 1998) will take many times longer on similar computer equipment. It is not clear whether modern computing power is sufficient to build the kind of model that appears to be needed. We return to this issue in a later section.

## DEVELOPING A NEW MODEL OF BACKWARD MASKING

Since a new kind of model appears to be needed, this is a good opportunity to consider the desired properties and features of such a model. The development of such a model needs to be constrained by both what is technically possible and also by what will be of interest to other researchers.

The last point deserves elaboration. Although there are many models of backward masking, they are used almost exclusively by modellers themselves. These uses include demonstrations of how the models match experimental data, tests of model assumptions, promotion of model development, comparing and contrasting models, and (rarely) identifying new properties of masking that are predicted by the models. Significantly, the models have almost never been used to explain other aspects of cognition, perception, or consciousness. This is notable because masking techniques are often used to experimentally investigate these topics. Apparently, the properties and features of current models are not sufficient to contribute to the discussion of those topics. This lack of model use is not a healthy arrangement for the field. Ideally, non-modellers would use the models to explore aspects of cognition and introduce new ideas that would drive model development.

So what would a new model of masking ideally look like? Given the problems with the current models described above, the new model must combine models of spatial vision and models of temporal vision. Some of these model parts may already exists, but putting them together may not be trivial. In particular, models of spatial vision simply may not work properly when temporal dynamics are considered.

There is a tendency for scientists to want simple models, but a system that mathematically deals with both spatial and temporal aspects of visual perception is unlikely to be simple. There may be simple parts of the model and there may be principles that guide the main computations of the model, but the most interesting parts of perception will involve interactions between the simple model parts. When such interactions involve feedback and non-linear relationships, the resulting behaviour is unlikely to be simple. Indeed, past research indicates that there may be no way to predict the behaviour of such a system except by direct simulation. In this respect, the model will have to be studied in a way that is similar to psychophysical studies of human perception. Researchers will have to identify simulation experiments that test the behaviour of the system. This is a different view of modelling than most psychologists imagine. For most psychologists the definition of the model is essentially the same thing as understanding the model. In this different view though, one can define a model without fully understanding its behaviour.

**Figure 3.**
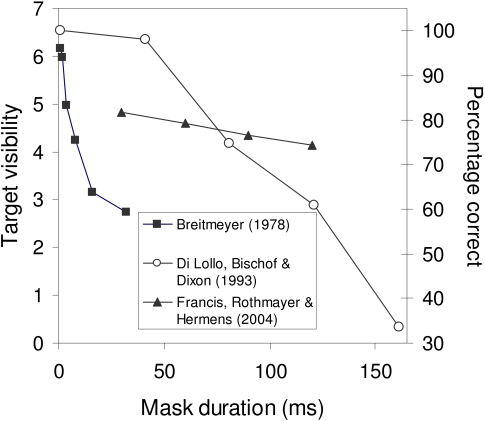
Robust effects of mask duration on masking. Even though there are substantive differences in the experiments, all of these studies show that masking grows stronger with increases in mask duration.

There is a risk that a research project like this may end up with a model that is just as complicated as what it hopes to explain. How should model behaviour be connected to experimental data in a way that clarifies our understanding of human perception and cognition? One useful line of investigation concerns robustness of behaviour. A robust behaviour is one that occurs for a variety of circumstances. For example, a robust experimental finding of backward masking is that increases in the duration of the mask tend to lead to stronger masking. This is true for a wide variety of stimuli, experimental tasks, observers, and other details of an experiment. Figure 3 summarizes experimental data from three very different studies that all demonstrate the effect of mask duration.

Here, we briefly describe the experiments because it helps to demonstrate how some masking effects exist across a variety of contexts and tasks. Breitmeyer (1978) had observers vary the luminance of a comparison stimulus to match the perceived brightness of a target disk that was masked by a surrounding annulus. The experiment varied the SOA between target and mask and varied the duration of the mask. Figure 3 plots target visibility for varying mask durations averaged across the various SOAs. In this experiment there is a sharp drop in target visibility as mask duration increases. Di Lollo, Bischof, and Dixon (1993) had observers report the orientation of a gap that was placed on one side of a target outline square. The mask was an outline square with a gap on each side. They kept the SOA at zero, but varied the mask duration. Again, Figure 3 shows that there is a drop in percentage correct as mask duration increased. Francis, Rothmayer, and Hermens (2004) had observers report the orientation of a target half disk among three distracting full disks. The mask was a set of annuli that surrounded the target and distracter elements. SOA, target duration, and mask duration were all varied. Figure 3 shows the effect of mask duration averaged across all SOAs and two target durations. Although the slope is more shallow than for the other data sets, again percentage correct decreases as mask duration increases. Although it also used a variety of mask durations, the study by Macknik & Livingstone (1998) is not included in this figure because they normalized the overall strength of masking for each stimulus condition. This normalization prevents a comparison of masking strength for different mask durations.

There may be several different ways to account for this robust experimental finding, but a key point is that it is robust. It holds for a variety of experimental tasks, stimuli, and contexts. Thus, whatever the hypothesized model mechanisms, the model behaviour must also be robust. That is, small variations in model parameters might change the magnitude of masking, but should not change the overall effect of increases in mask duration. Robust experimental findings should be explained by robust properties of the model.

**Figure 4.**
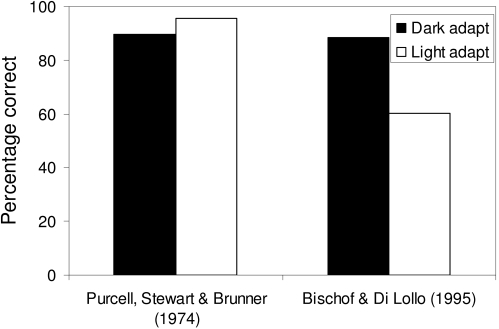
Sensitive effects of dark and light adaptation on masking. In one study, masking is stronger with dark adaptation than with light adaptation. In the other study just the opposite was found. The small quantitative differences in the Purcell et al. (1974) data relative to that of Bischof & Di Lollo (1995) reflects differences in the experimental task rather than the strength of adaptation. Both findings were highly significant from a statistical point of view.

Just the opposite is true for sensitive behaviours. For example, backward masking studies have found different effects of dark adaptation. Purcell, Stewart, and Bruner (1974) found that masking was stronger when observers were dark adapted. The data in Figure 4 are averaged across several SOAs. In contrast, Bischof and Di Lollo (1995) found that masking was absent when observers were dark adapted, but strong when observers were light adapted. The data in Figure 4are from the faintest stimuli in each condition, averaged across many SOAs. Both studies appear to be conducted properly, so the conclusion is that the effect of dark adaptation is sensitive to many details of the task, stimuli, observers, and other experimental conditions. As a result, a model’s explanation of the effect of dark adaptation needs to be similarly sensitive. In such a model, one would expect that changes in model parameters would lead to rather different model behaviours with regard to light adaptation.

In general, robust experimental findings can be used to identify the main structure and properties of a model. Such findings are not so effective at identifying the particular parameters that define the model’s behaviour. In contrast, sensitive experimental findings can be used to precisely parameterize a model, but tend to not be useful for characterizing the general structure and function of a model.

## MODEL STRUCTURE AND COMPUTATION

When constructing a model, one has to consider the units and mechanisms that make up the model components. Because backward masking is a tool that is used both by psychologists to explore aspects of human behaviour and by neuroscientists to explore properties of the brain, the ideal model will be defined in terms of neural units.

Ideally, the model would receive spatial images (as on a computer monitor) with an explicit representation of time. This arrangement would allow the model to essentially act as a “subject” in a psychophysical or neurophysiological experiment. There are good starting points for the development of this aspect of the model structure (e.g., Grossberg, 1997), although it is unclear whether current computing power is sufficient to provide the spatial and temporal resolution that appears to be needed to emulate a backward masking experiment.

Some quick calculations explain why there may be a problem finding sufficient computing power. The temporal model of Weisstein (1972) utilizes only six model neurons. On a PC running at 3.2 GHz with 1 GB of RAM, the simulation described in Francis (2003b) takes approximately 29 milliseconds to compute each point in a masking function (there is some variability because it depends on the SOA). A masking function curve such as in Figures 1 or 2 involves calculation of around 10-20 points. This means that such a curve will take between 290 and 580 milliseconds (plus a bit more for setting up the simulation and saving results). As an approximation, let us say the simulation time to produce a masking function curve is around 500 milliseconds. This is generally fast enough that a researcher can explore the model for variations of parameters and fits to experimental data.

The Weisstein model contains no representation of the spatial properties of the target or mask stimuli. Suppose that the model is extended in to 2-dimensional space by replicating the current model cells at multiple pixel locations. If the simulation grid is 200 by 200 pixels that each operate as the original model, this means that there are 200 × 200 = 40,000 pixels. To compute a masking function curve with this spatio-temporal Weisstein model would require 40,000 × 500 ms = 20,000,000 ms = 5.6 hours. Such a long time to compute a single masking curve is perhaps close to the limit of what would allow a researcher to explore a variety of model parameters.

A similar point can be seen by observing the computational requirements of a detailed spatial model of visual perception. Koch and Walther (2006) produced a MatLab version of the Itti et al. (1998) model of visual perception and have made their code available on the Internet. This model involves many spatial filters that are sensitive to different orientations, colours, and spatial scales. On the same computer as described above, this program took around ten seconds to compute the model’s response to an image of 700 by 560 pixels. The precise computation time depends on the properties of the image, but ten seconds is a ball park figure. If this model were extended to include a temporal component and the same computations were carried out every 50 milliseconds of real time, it would take 0.28 hours to go through one second of simulated time, which is approximately the duration of a single backward masking trial. A masking curve with 20 data points would require at least 5.6 hours of computation time.

The main point is that moving from a model of temporal vision or a model of spatial vision to a spatio-temporal model involves an enormous increase in computational requirements. Of course, faster computers and software compilers exist that could speed up the simulation times. On the other hand, it is very likely that translating either a temporal or spatial model of visual perception in to a spatio-temporal model will require new model components that will further increase the computational load of simulations.

### Feed forward and feedback models

There has been substantial discussion, both within the field of masking and elsewhere, about the importance of feedback within models. Some researchers have taken the stand that certain experimental findings rule out feed forward models (Di Lollo, Enns, & Rensink, 2000, 2002). This topic deserves some additional discussion because, contrary to common belief, such debates rarely help drive model development. A system with feedback may behave exactly the same as a feed forward system.

Part of the confusion is due to people failing to make a distinction between anatomical feedback and computational feedback. Neurophysiologists have established that there are re-entrant fibres that project from higher cortical areas to lower cortical areas. This is an established anatomical fact, and it is quite likely that these fibres influence perceptual experience. Exactly what these signals do is less clear. For psychologists, though, the behaviour of the system is more important than the anatomy. Currently there is no known model behaviour that can be used as a “marker” for feedback.

**Figure 5.**
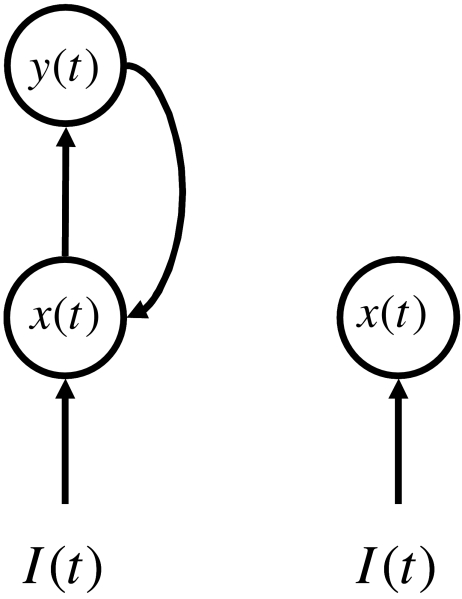
Two hypothetical systems that differ in whether they have anatomical feedback connections (left) or not (right). See the text for a mathematical model of how such systems might behave.

Worse still, there is no clear connection between anatomical feedback and mathematical equations. Consider the two different anatomical systems in Figure 5. The system on the left has anatomical feedback while the system on the right does not. The circles can be thought of as neurons or populations of neurons; the details are not so important for the current discussion. Because we are interested in the dynamics of perception, it is natural to describe the “activity” of the units with differential equations that describe the instantaneous changes in activity. The feedback system might be described with a pair of differential equations:

(1) $$\frac{dx(t)}{dt}=-Ax(t)+I(t)+By(t)$$

and

(2) $$\frac{dx(t)}{dt}=-Cy(t)+Dx(t)$$

Here, the capital letters indicate parameters and the terms –*Ax(t)* and –*Cy(t)* indicate passive decay. The activity from the higher level, *y(t)*, feeds back in to the equation for activity at the lower level, *x(t)*, through the term *By(t)*. In this case, the mathematical layout of terms appears to match the anatomical structure.

For the feed forward system on the right there might be only one equation.

(3) $$\frac{dx(t)}{dt}=-Fx(t)+I(t)$$

The term –*Fx(t)* again indicates passive decay and there is no feedback from higher areas.

Now let us add one further condition to the system. Suppose the differential equation at the higher stage of the feedback system runs much faster than the differential equation of the lower stage. (This would be the case if *C* and *D* are much larger than *A* and *B*.) In this situation the value of *y(t)* changes dramatically while *x(t)* is approximately constant. The value *y(t)* can be treated as its algebraic equilibrium value (found by setting equation 2 equal to zero and solving for *y(t)*):

(4) $$y(t)=\frac{D}{C}x(t)$$

This has a significant effect on how we can describe the rest of the feedback system. If we replace *y(t)* in equation (1) with the right hand side of equation (4), we get

(5) $$\frac{dx(t)}{dt}=-Ax(t)+I(t)+B\frac{D}{C}x(t)$$

Now define the parameter

(6) $$F=A-B\frac{D}{C}$$

If we combine the terms in equation (5) that multiply *x(t)*, the equation becomes

(7) $$\frac{dx(t)}{dt}=-Fx(t)+I(t)$$

This is identical to equation (3)! In this case the behaviour of *x(t)* is mathematically identical in the feedback system and in the feed forward system. Thus, even if the anatomy of the visual system provides clear evidence of re-entrant or feedback signals, this does not guarantee that the system behaves any differently than a feed forward system. It is noteworthy too that, at first glance, equation 7 would seem like a very poor description of the feedback system in Figure 5. In fact, though, it fully captures the behaviour of the lower unit and the behaviour of the upper unit is just a multiple of the lower unit.

Of course, such isomorphism may not always be possible or practical, but one never knows for sure what the feedback signals actually do, and there are many other analogous situations that blur the distinction between feedback and feed forward systems. As Reeves (this volume) observes, mathematicians have noted that any feedback system can be approximated by a suitably complex feed forward system.

None of this is to say that re-entry, feedback, and non-linearities should be not investigated. To the contrary, their presence in the anatomy of the nervous system suggests that they need to be characterized and studied carefully. The problem with many of the current discussions of feedback in masking is that they fail to specify the exact nature of re-entry feedback (Di Lollo et al., 2000; Enns, 2004). As a result there are no precise predictions about what the feedback actually does within the system.

On the other hand, when the feedback is characterized in a precise quantitative way, the resulting model can make very precise statements about how the system behaves and what different parts of the model are doing (e.g., Hansen & Neumann 2004; Raizada & Grossberg, 2001).

## USING A MODEL OF MASKING

Having identified what a quantitative model of backward masking might look like, we now turn to whether it should be built. The question is whether there is sufficient need for a model to justify the required effort and expense. In an attempt to answer affirmatively we can consider some possible uses of such a model.

1. *Create an ideal mask for a given target and task*. Backward masking is commonly used to study other aspects of cognition. At the moment the properties of the mask are found by experimental trial and error. Such work is frustratingly slow and inefficient. A good model might be able to speed up the process by identifying mask properties that would be able to mask the target properties most important to the experimenter.

2. *Identify new experimental techniques to explore consciousness*. Although backward masking has a long history of contributing to studies of consciousness there have always been concerns about what the studies are actually measuring. A computational model of masking might be able to identify new experimental studies that avoid some of the concerns with these techniques.

3. *Identify experimental and neurophysiological markers for mental disease*. Several studies have shown that backward masking differs for people with various types of mental disease, relative to normals (Braff & Saccuzzo, 1981; Green, Nuechterlein, & Mintz, 1994). A model may be able to help identify what mechanisms are different, which could lead to early detection and better understanding of how the disease operates.

Since backward masking is used as a tool to investigate many other neurophysiological and mental phenomenons, a good model would surely be useful in many other situations.

## CONCLUSIONS

Backward masking is an important topic that is used throughout psychology both to investigate visual perception and as a tool to study other aspects of cognition. Unfortunately, there is currently no theory of how backward masking operates that can guide researchers on how to use masking. In particular, all of the quantitative models of backward masking have recently been shown to be invalid because they lack a sufficient representation of visual space.

These findings suggest that new types of models of backward masking are needed. It seems that a new model needs to deal with both space and time so that it can work with visual stimuli that are similar to those used in psychophysical experiments. The model needs to be flexible enough to operate in a variety of experimental situations and be connected to many different perceptual tasks. The model needs to be described in neurophysiological terms. The model needs to be structured in such a way that it can be used by non-modelers. Finally, the model needs to be able to make particular predictions of neurophysiological and mental behaviour so that it can be tested and developed in a meaningful way.
